# Development and validation of a rabbit model of *Pseudomonas aeruginosa* non-ventilated pneumonia for preclinical drug development

**DOI:** 10.3389/fcimb.2023.1297281

**Published:** 2023-12-11

**Authors:** Emmanuelle Gras, Trang T. T. Vu, Nhu T. Q. Nguyen, Vuvi G. Tran, Yanjie Mao, Nguyen D. Tran, Nam H. Mai, Oliver X. Dong, David H. Jung, Natalia L. P. P. Iorio, Helvecio C. C. Povoa, Marcos Gabriel Pinheiro, Fabio Aguiar-Alves, William J. Weiss, Bo Zheng, Lily I. Cheng, Charles K. Stover, Bret R. Sellman, Antonio DiGiandomenico, Laure Gibault, Florent Valour, Binh An Diep

**Affiliations:** ^1^ Division of HIV, Infectious Diseases, and Global Medicine, Department of Medicine, University of California San Francisco, San Francisco, CA, United States; ^2^ Université François Rabelais, Tours, France; ^3^ Department of Orthopaedic Surgery, Shanghai Jiao Tong University Affiliated Sixth People’s Hospital, Shanghai, China; ^4^ University of Medicine and Pharmacy, Ho Chi Minh City, Vietnam; ^5^ Department of Basic Science, Fluminense Federal University, Nova Friburgo, Rio de Janeiro, Brazil; ^6^ Pathology Program, Universidade Federal Fluminense, Niterói, Rio de Janeiro, Brazil; ^7^ Pre-Clinical Services at UNT Health Science Center, Fort Worth, TX, United States; ^8^ Clinical Pharmacology & DMPK, AstraZeneca, Gaithersburg, MD, United States; ^9^ Early Vaccines and Immune Therapies, AstraZeneca, Gaithersburg, MD, United States; ^10^ Pathology Department, George Pompidou European Hospital, Paris, France; ^11^ Department of Infectious Diseases, Hospices Civils de Lyon, Lyon, France; ^12^ CIRI – Centre International de Recherche en Infectiologie, Inserm, U1111, University of Lyon, Lyon, France; ^13^ Université Claude Bernard Lyon 1, CNRS, UMR5308, Ecole Normale Supérieure de Lyon, University of Lyon, Lyon, France

**Keywords:** non-ventilated pneumonia model, preclinical efficacy model validation, rabbit model, *Pseudomonas aeruginosa*, meropenem, tobramycin

## Abstract

**Background:**

New drugs targeting antimicrobial resistant pathogens, including *Pseudomonas aeruginosa*, have been challenging to evaluate in clinical trials, particularly for the non-ventilated hospital-acquired pneumonia and ventilator-associated pneumonia indications. Development of new antibacterial drugs is facilitated by preclinical animal models that could predict clinical efficacy in patients with these infections.

**Methods:**

We report here an FDA-funded study to develop a rabbit model of non-ventilated pneumonia with *Pseudomonas aeruginosa* by determining the extent to which the natural history of animal disease reproduced human pathophysiology and conducting validation studies to evaluate whether humanized dosing regimens of two antibiotics, meropenem and tobramycin, can halt or reverse disease progression.

**Results:**

In a rabbit model of non-ventilated pneumonia, endobronchial challenge with live *P. aeruginosa strain* 6206, but not with UV-killed Pa6206, caused acute respiratory distress syndrome, as evidenced by acute lung inflammation, pulmonary edema, hemorrhage, severe hypoxemia, hyperlactatemia, neutropenia, thrombocytopenia, and hypoglycemia, which preceded respiratory failure and death. Pa6206 increased >100-fold in the lungs and then disseminated from there to infect distal organs, including spleen and kidneys. At 5 h post-infection, 67% of Pa6206-challenged rabbits had PaO_2_ <60 mmHg, corresponding to a clinical cut-off when oxygen therapy would be required. When administered at 5 h post-infection, humanized dosing regimens of tobramycin and meropenem reduced mortality to 17-33%, compared to 100% for saline-treated rabbits (*P*<0.001 by log-rank tests). For meropenem which exhibits time-dependent bactericidal activity, rabbits treated with a humanized meropenem dosing regimen of 80 mg/kg q2h for 24 h achieved 100% T>MIC, resulting in 75% microbiological clearance rate of Pa6206 from the lungs. For tobramycin which exhibits concentration-dependent killing, rabbits treated with a humanized tobramycin dosing regimen of 8 mg/kg q8h for 24 h achieved C_max_/MIC of 9.8 ± 1.4 at 60 min post-dose, resulting in 50% lung microbiological clearance rate. In contrast, rabbits treated with a single tobramycin dose of 2.5 mg/kg had C_max_/MIC of 7.8 ± 0.8 and 8% (1/12) microbiological clearance rate, indicating that this rabbit model can detect dose-response effects.

**Conclusion:**

The rabbit model may be used to help predict clinical efficacy of new antibacterial drugs for the treatment of non-ventilated *P. aeruginosa* pneumonia.

## Introduction

The prevalence of antibiotic resistance in Gram-negative bacteria, especially *Pseudomonas aeruginosa, Acinetobacter baumannii* and *Klebsiella pneumoniae*, is alarming (World Health Organization, 2017). Although effective antibacterial drugs are still available, there is an urgent need for renewing the current pipeline. New antibacterial drugs with activity against a single species, or those potentiating activity of existing antibiotics, are urgently needed, but clinical trials may be challenging as the target species may be an infrequent cause of human disease or patients often receive pre-study and concomitant antibacterial therapy (Byrne et al., 2020). Development of new antibacterial drugs is facilitated by preclinical animal models that could predict clinical efficacy in patients with difficult-to-treat infections, including hospital-acquired non-ventilated bacterial pneumonia and ventilator-associated bacterial pneumonia (HABP/VABP) indications.

A systematic review performed by the U.S. Food and Drug Administration (FDA) identified 180 studies of animal models of bacterial pneumonia used in regulatory submissions for investigational new drugs (INDs) to the Division of Anti-Infectives from 2000 to 2019 (Waack et al., 2020). The vast majority (177/180) of these studies used mouse and rat pneumonia models for testing new antimicrobial agents targeting two Gram-positive (*Staphylococcus aureus* and *Streptococcus pneumoniae*) and seven Gram-negative organisms (*Klebsiella pneumoniae*, *Haemophilus influenzae*, *Stenotrophomonas maltophilia*, *Escherichia coli*, *Enterobacter cloacae*, *Acinetobacter baumannii*, and *Pseudomonas aeruginosa*), with only three studies using a rabbit pneumonia model for targeting *S. pneumoniae* (Waack et al., 2020). None of these 180 studies utilized an animal model of ventilator-associated pneumonia.

Rodent models have proven advantageous for drug discovery because of their relatively low cost, which facilitates their use in high throughput screening and for initial efficacy testing (Andes and Craig, 2002; Ambrose et al., 2007). For bridging efficacy data from rodent preclinical studies to human clinical trials, other animal species are used. Non-human primate models, primarily rhesus macaques, have been used traditionally for this purpose but, like mice, these Old World monkeys are quite resistant to some Gram-negative infections, including those caused by *P. aeruginosa* infection (Saslaw et al., 1972), which could be due to their innate resistance to lipopolysaccharide (LPS) (Warren et al., 2010). In contrast, rabbits are very similar to humans and chimpanzees in their susceptibilities to LPS (Mathison et al., 1988; Vaure and Liu, 2014) and they are naturally susceptible to *P. aeruginosa* infections (O'Donoghue and Whatley, 1971).

Responding to a critical need, the FDA awarded several research contracts to enable the development and refinement of animal models of serious infections, including pneumonia, caused by *P. aeruginosa* and *A. baumannii* that can be used to predict whether candidate antibacterial drugs will be efficacious in humans. Two of these FDA awards were for the development of a rabbit model of ventilator-associated pneumonia with *P. aeruginosa*, which we described recently (Nguyen et al., 2021), and a rabbit model of non-ventilated pneumonia with *P. aeruginosa*, which we described herein. Our complementary rabbit ventilated and non-ventilated pneumonia models reproduced different pathophysiology and phenotypic characteristics of the diverse range of lung infections observed in humans (Zilberberg et al., 2022). Furthermore, humanized dosing regimens of meropenem and tobramycin proved efficacious in the rabbit non-ventilated pneumonia model with *P. aeruginosa*, thereby illustrating the potential utility of this model for preclinical drug development.

## Methods

### Study design

We selected *P. aeruginosa* strain 6206 (Pa6206) for the rabbit model because of its virulence in mouse models and its susceptibility to all available anti-pseudomonal antibiotics (i.e. minimum inhibitory concentration, MIC, of 0.25 µg/mL for meropenem and 1.0 µg/mL for tobramycin) (Cole et al., 2003; Priebe et al., 2003; DiGiandomenico et al., 2014; Thanabalasuriar et al., 2017). Firstly, we conducted a natural history study using 36 outbred, female and male, New Zealand white rabbits (six animals each euthanized at 3, 4, 5, 6, 10 h post-infection, and another six animals monitored until their terminal endpoint) to characterize the evolution of non-ventilated pneumonia caused by Pa6206 and to define an optimal time window for therapeutic intervention. Experimental endpoints for the natural history study included lung weight-to-body weight (LW/BW) ratios, bacterial counts, clinically relevant biomarkers, including complete blood count, blood gas, metabolic panel, plasma interleukin 8 (IL-8, CXCL-8), and gross and histopathology of the lungs. Seven additional rabbits, euthanized at 5 or 96 h after endobronchial challenge with either lactated Ringer’s solution or *Pa*6206 killed by irradiation with ultraviolet (UV) light, were included as controls to show that these rabbits did not develop non-ventilated pneumonia. Secondly, PK studies in uninfected and infected rabbits (n=3 per group) were performed to define humanized dosing regimens for meropenem and tobramycin. These two antipseudomonal drugs have different clinical pharmacokinetic/pharmacodynamic (PK/PD) drivers of efficacy, i.e., the percentage of the dosing interval that drug concentration remains above the MIC (%T>MIC) for meropenem and peak concentration/minimum inhibitory concentration (C_max_/MIC) for tobramycin (Ambrose et al., 2007; Kalil et al., 2016). Lastly, two therapeutic randomized, placebo-controlled studies were conducted with 24 and 36 outbred, female and male, New Zealand white rabbits (n=12 per experimental group). In the first treatment study, starting at 5 h post-infection, rabbits were administered IV tobramycin at 5 mg/kg q8h for 24 h, or placebo with equivalent volume of saline q8h for 24h. In the second treatment study, starting at 5 h post-infection, rabbits were administered 80 mg/kg meropenem IV q2h for 24h, 2.5 mg/kg tobramycin IV once followed by equivalent volume of saline q2h for 24h, or placebo with equivalent volume of saline q2h for 24h. The primary endpoint was overall mortality from acute respiratory failure. Secondary endpoints included bacterial burden (log_10_CFU/organ) and LW/BW ratios.

### *P. aeruginosa* strain and inoculum preparation

The pan-susceptible Pa6206, encoding the type III secreted toxin *exoU*, was used for infection. This LPS-smooth serogroup O11 strain, isolated from a corneal infection (Bascom-Palmer Eye Institute, Miami, Florida), is used by many laboratories in animal models (Thanabalasuriar et al., 2017). It was acquired originally from J.B. Goldberg (University of Virginia, Charlottesville, VA). MICs for meropenem and tobramycin were determined using the microbroth dilution methods described by the Clinical and Laboratory Standards Institute 2012.

Inocula were prepared from the Pa6206 stock in cryotubes in a -80°C freezer as previously described (Le et al., 2018). In brief, the strain was streaked for colony isolation onto 5% sheep blood agar plate, and 5-10 well-isolated colonies were inoculated in 12 mL of tryptic soy broth (TSB) in a 50-mL vented-cap tube with shaking at 150 RPM and 37°C for 16 to 20 h. The overnight culture (60 µl) was then transferred to 12 mL of fresh TSB and incubated with shaking at 150 RPM and 37°C for 12 h, to an optical density at 600 nm (OD_600nm_) between 1.7 and 1.8. Bacteria were collected by centrifugation at 16°C, washed once, and then resuspended by vortexing in lactated Ringer’s solution (LRS). The washed cells were then diluted in LRS to an OD_600nm_ of 1.455 to 1.465, corresponding to 1.2 x 10^9^ CFU/ml. This stock solution of bacteria was further diluted in LRS to the desired concentration of 1-2 x 10^8^ CFU/mL and was confirmed by serial dilution on 5% sheep blood agar plates. The final inoculum was used for rabbit infection within 1 h. A portion of the live Pa6206 inoculum was also transferred to a round sterile plastic bowl containing a sterile magnetic stir bar and exposed to 60 min of irradiation using a germicidal UV-C lamp (TUV PL-L 95W/4P HO, Philips) at room temperature while under constant stirring. 100 µL of the UV-exposed bacterial suspension was plated onto 5% blood sheep agar, which showed no bacterial growth.

### Rabbit non-ventilated pneumonia model

The rabbit non-ventilated pneumonia model was reviewed and approved by the University of California San Francisco Institutional Animal Care and Use Committee. Experiments were conducted in a facility certified by the Association for Assessment and Accreditation of Laboratory Animal Care International. Outbred, female and male, New Zealand White rabbits (8 to 12 weeks-old, 2-3 kg, Western Oregon Rabbit Co.) arrived at least two days prior infection for acclimation. They were housed individually in stainless-steel cages in a climate-controlled housing room with 12-hours alternative light and dark cycles. They had unlimited access to food pellets and water, supplemented twice daily with fresh fruits and vegetables. Clinical parameters (body weight, temperature, activity) were assessed to ensure normal adaptation to their new environment. All blood samples and IV injections were done after local anesthesia using 5% lidocaine cream. Blood samples were taken from the ear artery and IV injections were administered through the marginal ear vein. On infection day, animals were infected according to the previously described experimental procedure (Le et al., 2018). Briefly, 1.8-mL instillation containing Pa6206 was delivered directly into the lungs of anesthetized rabbits through a 2.5-mm pediatric endotracheal tube that was immediately removed after instillation of the bacterial inoculum. After infection, animals were put back in their cages and clinically evaluated every two hours for the first 36 hours and then three times per day up to 96 h post-infection. At predetermined time points, animals were euthanized by intravenous injection with a lethal dose of sodium pentobarbital. During each clinical evaluation, rabbits with respiratory failure, defined as increased respiratory rate to >75 breadths/min, cough and cyanosis, and confirmed with a blood lactate test exceeding 10 mmol/L, were euthanized by intravenous injection with a lethal dose of sodium pentobarbital. Lungs, spleen and kidneys were harvested aseptically, photographed and weighed. Bacterial counts were determined after homogenization of 3-4 pieces (totaling 0.2-0.3 g) from different parts of each organ in 1 mL of 0.9% saline solution by serial dilution on 5% sheep blood agar plates.

### Preparation and administration of meropenem and tobramycin for bolus intravenous infusion

Meropenem (500 mg) was reconstituted in 10 ml normal saline to 50 mg/mL and administered by bolus IV injection through the rabbit marginal ear vein at 80 mg/kg every 2 h. Tobramycin (40 mg/mL) was diluted in normal saline to 4 mg/mL and administered by bolus IV injection through the marginal ear vein at 2.5 mg/kg once or 5 mg/kg every 8 h.

### Pharmacokinetic studies

PK parameters were determined in uninfected and infected rabbits (3-5 per group) for both meropenem and tobramycin. To determine meropenem PK, serial blood samples were collected from the central ear arteries at 0, 5, 30, 55, 80, and 120 min after a single intravenous dose of 80 mg/kg meropenem for uninfected rabbits and at 0, 5, 30, 60, 90 and 120 min after the 12^th^ (and last) dose of 80 mg/kg meropenem for infected rabbits. To determine tobramycin PK in uninfected and infected rabbits, serial blood samples were drawn at 0, 0.5, 1, 2, 4, 8, and 24 h after a single intravenous dose of 2.5 mg/kg tobramycin. Plasma concentrations of meropenem and tobramycin were determined by liquid chromatography-tandem spectrometry (LC-MS/MS) and entered into PKSolver 2.0 to calculate pharmacokinetic parameters using the module for one-compartment analysis of plasma data after intravenous bolus input (Zhang et al., 2010). The lower limit of quantification was 0.19 µg/mL for tobramycin and 0.024 µg/mL for meropenem in rabbit plasma.

### Histology

**Figure 1:**
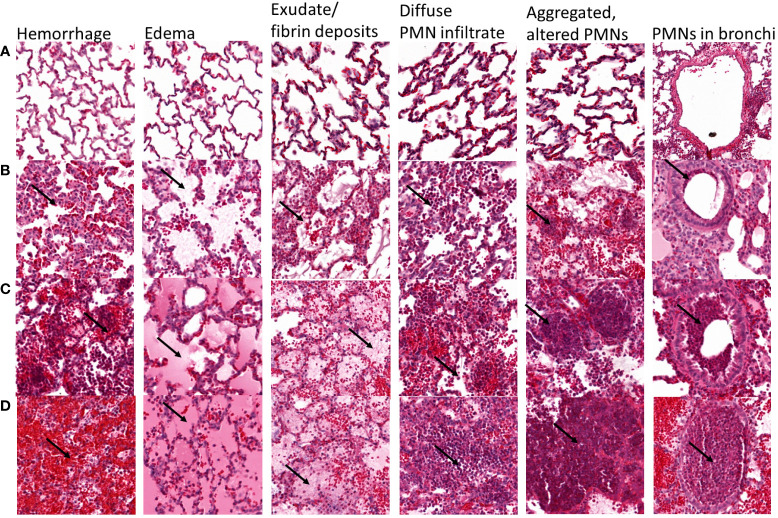
Representative lung sections showing semi-quantitative scoring of the severity of hemorrhage, edema, exudate/fibrin deposits, diffuse PMN infiltrate, multifocal aggregated and altered PMNs, and PMNs in bronchi. Each lung section was scored according to the following scale: A = 0, absent; B = 1, mild; C = 2, moderate; D = 3, severe. For hemorrhage, arrows show red blood cells, and **(A)** absent, **(B)** scarce and located near the alveolar septa, **(C)** responsible for a mild and diffuse infiltration with patchy aggregates, and **(D)** diffuse massive infiltrate covering >50% of the lung section. For edema, arrows point to edema, and **(A)** absent, **(B)** mild streaked edema, **(C)** abundant edema filling partially the alveoli in 30-50% of the lung section, **(D)** abundant edema entirely filling the alveoli in >50% of the lung section. For exudate/fibrin, arrows point to fibrin, **(A)** absent, **(B)** mild fibrin exudate mixed with edema, **(C)** moderate fibrin exudate covering 30-50% of the lung section, **(D)** patchy fibrin exudate covering >50% of the lung section. For diffuse PMN infiltrates, arrows point to PMN, and **(A)** absent, **(B)** scarce PMN infiltrate with large unscathed areas, **(C)** mild PMN infiltrate covering the majority of the section with spared areas, **(D)** massive intra-alveolar PMN infiltrate covering >50% of the lung section. For multifocal aggregated, altered PMNs, arrows point to PMNs, **(A)** absent, **(B)** scarce small PMN aggregates, **(C)** moderately aggregated and altered PMN, **(D)** multiple big PMN aggregates covering the greater part of the lung section. For PMNs in bronchi, arrows point to PMNs, **(A)** absent, **(B)** PMN underlining the bronchiole/bronchi wall, **(C)** occupying >50% of the bronchiole/bronchi wall, **(D)** fulfilling the entire bronchiole/bronchi section.

Immediately after death or euthanasia, the left lung was harvested and fixed by gravity instillation through the mainstem bronchus with 10% formalin. For each left lung, a single section from the upper lobe and a single section from the lower lobe were stained with haematoxylin and eosin (H&E) and analyzed in a semiquantitative manner by a single trained physician (E.G.) blinded to the experimental groups. **Figure 1** shows lung sections for each level of hemorrhage, edema, exudate/fibrin, diffused PMN infiltrate, multifocal aggregated and altered PMNs, and PMN in bronchi according to a semi-quantitative severity scoring scheme: A = 0, absent; B = 1, mild; C = 2, moderate; D = 3, severe. **Table 1** shows the detailed results for this semi-quantitative analysis.

**Table 1:** Longitudinal lung histological analysis for the rabbit natural history study based on semiquantitative scoring.

	Hemorrhage	Edema	Exudate/fibrin	Diffuse PMN infiltrate	multifocal altered PMN aggregates	PMN in bronchi (bronchiolitis)
Vehicle (LRS) control	**1** (1/1)	**0** (0/0)	**0** (0/0)	**0** (0/0)	**0** (0/0)	**0** (0/0)
UV-killed Pa6206 control at 5 h post-infection	**1** (1/1, 1/1, 1/1)	**0** (0/0, 0/0, 0/0)	**0** (0/0, 0/0, 0/0)	**1** (1/1, 1/1, 1/1)	**0** (0/0, 0/0, 0/0)	**0** (0/0, 0/0, 0/0)
UV-killed Pa6206 control at 96 h post-infection	**1** (1/1, 1/1, 1/1)	**0** (0/0, 0/0, 0/0)	**0.2** (0/0, 0/0, 0/1)	**1** (1/1, 1/1, 1/1)	**0.3** (0/0, 0/0, 1/1)	**0.3** (0/0, 0/0, 1/1)
llive Pa6206 challenge at 3 h post-infection	**1.1** (1/2, 0/1, 1/1, 1/1, 1/2, 1/1)	**0.3** (1/0, 0/0, 0/1, 0/0, 1/1, 0/0)	**0.4** (1/1, 0/1, 0/1, 0/0, 0/1, 0/0)	**1.3** (2/2, 1/1, 1/1, 1/1, 1/3, 1/1)	**0.8** (0/2, 0/0, 2/1, 1/0, 1/2, 0/0)	**0.5** (0/0, 1/0, 1/0, 0/1, 1/1, 0/1)
live Pa6206 challenge at 4 h post-infection	**1.2** (1/2, 1/2, 1/1, 1/1, 1/1, 1/1)	**1.3** (2/1, 1/2, 2/2, 1/1, 1/1, 1/1)	**1** (1/2, 1/1, 1/1, 1/1, 1/1, 1/0)	**1.4** (2/1, 1/1, 2/1, 1/2, 2/1, 1/2)	**1.3** (2/2, 1/1, 1/2, 1/2, 1/1, 1/1)	**1** (1/1, 0/1, 1/1, 1/1, 1/1, 2/1)
live Pa6206 challenge at 5 h post-infection	**1.5** (1/1, 1/1, 2/3, 2/1, 1/1, 1/3)	**1.3** (2/1, 0/1, 2/2, 2/2, 1/1, 1/1)	**1.1** (1/1, 0/1, 2/0, 1/1, 1/2, 1/2)	**1.5** (2/2, 1/1, 2/1, 1/2, 1/1, 2/2)	**1.2** (1/1, 0/0, 2/0, 1/2, 2/2, 2/1)	**0.9** (0/1, 0/1, 0/1, 0/1, 1/1, 3/2)
live Pa6206 challenge at 6 h post-infection	**1.3** (1/2, 1/1, 1/1, 1/1, 2/2, 2/1)	**1.5** (1/2, 0/2, 1/2, 1/1, 1/1, 3/3)	**1.2** (1/2, 0/1, 1/2, 0/1, 2/2, 1/1)	**2** (2/2, 1/2, 2/2, 2/2, 3/3, 1/2)	**1.2** (1/2, 0/1, 0/1, 1/1, 2/3, 1/1)	**1.3** (2/2, 0/1, 1/0, 2/2,1/2, 1/2)
live Pa6206 challenge at 10 h post-infection	**1.7** (2/1, 2/2, 1/2, 2/1, 2/2, 2/1)	**1.8** (2/2, 2/2, 1/2, 1/1, 1/3, 2/2)	**1.5** (2/1, 1/2, 1/1, 2/2, 1/2, 2/1)	**1.8** (2/1, 1/3, 1/2, 2/2, 2/2, 2/1)	**2.2** (2/2, 2/3, 1/2, 2/3, 2/3, 2/2)	**1.4** (2/2, 2/1, 0/1, 2/2, 2/2, 0/1)
live Pa6206 challenge at terminal endpoint	**2.5** (2/2, 2/2, 3/3, 3/3, 2/3, 3/2)	**2.5** (2/2, 2/2, 3/3, 2/2, 3/3, 3/3)	**2.1** (2/2, 2/2, 2/3, 2/2, 2/2, 3/1)	**2.3** (2/3, 2/2, 3/3, 2/2, 2/2, 3/2)	**2.3** (1/3, 2/2, 1/3, 2/3, 3/3, 3/2)	**1.7** (1/2, 1/1, 3/3, 0/0, 3/3, 2/1)

Mean values (bold font) of individual pathology scores within parenthesis for the rabbits used for each experimental time points are shown. Two lung sections from each rabbit were analyzed independently and their respective scores are separated by a forward slash “/”. Lungs from one rabbit challenged with vehicle (lactated Ringer’s solution, LRS) and 6 rabbits challenged with UV-killed Pa6206 were included for comparison. H&E-stained lung sections were scored for evidence of hemorrhage, edema, exudate/fibrin, diffuse PMN infiltrate, aggregated and altered PMN, and PMN in bronchi according to the following scale: 0 = absent; 1 = mild; 2 = moderate; 3 = severe as detailed in **Figure 1**.

### Blood gas, metabolic panel, and hematology analysis

Blood biomarkers were performed for the natural history of infection study at 0, 3, 4, 5, 6, 10 h post-infection and at time of death (13-21 h post-infection). The Element Point of Care Rapid Blood Analyzer^©^ (Heska, Loveland, CO) was used for analysis of blood gas (pH, partial pressure of oxygen [*p*O_2_] and of carbon dioxide [*p*CO_2_], base excess), electrolytes (sodium [Na^+^], potassium [K^+^], chloride [Cl^-^], ionized calcium [iCa^++^]) and chemistry parameters (creatinine, glucose, lactate). The Element HT5 Veterinary Hematology Analyzer^©^ (Heska) was used to determine white blood cells differential, red blood cells and platelets parameters. VetScan VS2^©^ (Abaxis, Union City, CA) enabled analysis of albumin, alkaline phosphatase (ALP), alanine aminotransferase (ALT), aspartate aminotransferase (AST), total protein (TP), amylase, total bilirubin, and blood urea nitrogen.

### Plasma IL-8 (CXCL-8) quantification

Sandwich ELISA kit was used for quantification of rabbit IL-8 concentrations in plasma samples (Raybiotech, Peachtree Corners, GA).

### Statistical analysis

For the natural history study, a sample size of 6 animals for each time point was needed because of rabbit-to-rabbit variability in their infection course and based on prior data from the rabbit *P. aeruginosa* non-ventilated pneumonia model (Le et al., 2018). Test for linear trend (GraphPad Software v9.0, San Diego, California, USA) was used to evaluate the hypothesis that the means of ARDS parameters (LW/BW ratio and biomarkers) tend to get higher (or lower) over time. For efficacy study, the predetermined sample size of 12 rabbits *per* experimental group yielded a power of 0.8 at *P*<0.025 (Bonferroni corrected *P*<0.025 threshold to account for two comparisons, e.g. saline *vs*. meropenem and saline *vs*. tobramycin) to detect an effect size/hazard ratio=0.32 (corresponding to 50% or greater difference overall survival) by one-sided log-rank test using the Schoenfeld method (STATA, version 10, College Station, TX). Survival curves were generated using the Kaplan-Meier method and compared between groups using the log rank (Mantel-Cox) test. For other study endpoints (CFU counts in lungs, spleen and kidneys, LW/BW ratios, biomarkers, etc.), normal distribution was not assumed, so groups were compared using a non-parametric one-way ANOVA with Kruskal-Wallis test followed by Dunn’s multiple comparisons test.

## Results

### Natural history of infection in rabbit model of *P. aeruginosa* non-ventilated pneumonia

**Figure 2:**
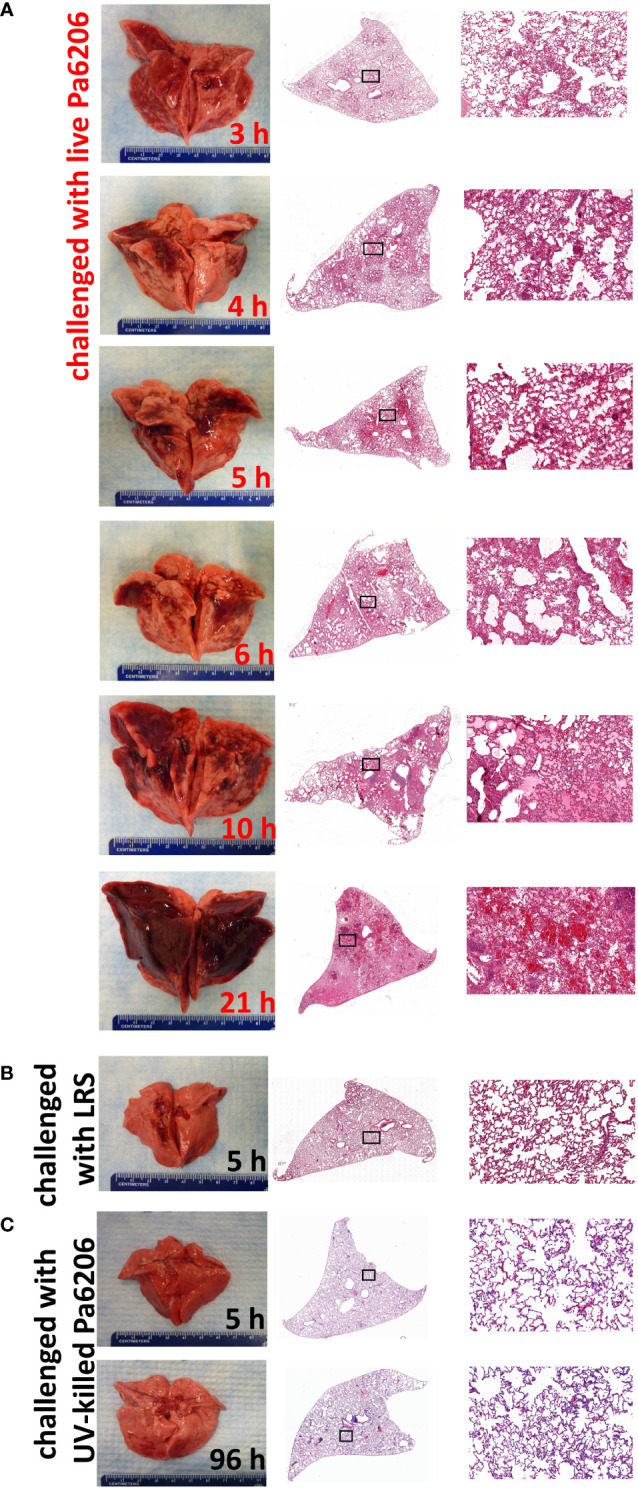
Longitudinal pathological changes in the lungs of rabbits with *P. aeruginosa*-induced non-ventilated pneumonia. Gross and histological changes of representative lungs harvested at **(A)** pre-determined time points of 3, 4, 5, 6, 10 h after endobronchial instillation with live Pa6206, or at 21 h after endobronchial instillation with live Pa6206 when rabbit developed respiratory failure, **(B)** 5 h after endobronchial instillation with LRS, and **(C)** 5 h or 96 h after endobronchial instillation with UV-killed Pa6206. Semi-quantitative scoring of the severity of hemorrhage, edema, exudate/fibrin, diffuse PMN infiltrate, multifocal altered PMN aggregates, and bronchiolitis are provided in **Table 1** and **Figure 3**.

To determine the extent to which the natural history of animal disease reproduces human pathophysiology as well as the time of onset of clinical and biological abnormalities and define the optimal time to start antibacterial treatment, we conducted a natural history study in which rabbits infected with 1.1 x 10^8^ CFUs of live Pa6206 were euthanized at pre-determined time points to evaluate changes in rabbit pathophysiology. Gross and histopathology showed a time-dependent increase in severity of pulmonary edema and hemorrhage in lungs from rabbits challenged with live Pa6206 (**Figure 2A**). Longitudinal histological analysis of infected lungs based on a semi-quantitative scoring system (**Figure 1**) showed a time-dependent increase in severity of hemorrhage, edema, exudate/fibrin, diffuse polymorphonuclear leukocyte (PMN) infiltrates, aggregated and altered PMNs, and PMNs in bronchi (**Table 1**). Progression of infection was associated with intra-alveolar edema and hemorrhage (**Figure 2A**; **Table 1**) as seen in the exudative phase of patients with acute respiratory distress syndrome (ARDS) (Thille et al., 2013). In contrast, endobronchial instillation with vehicle (lactated Ringer’s solution, LRS) or UV-killed Pa6206, which resulted in minimal alveolar hemorrhage and neutrophilic infiltration (**Figures 2B, C**; **Table 1**), did not result in progression to lethal pneumonia.

**Figure 3:**
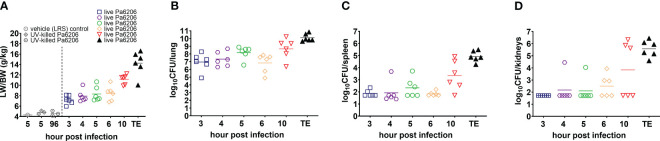
Natural history of *P. aeruginosa*-induced non-ventilated pneumonia in rabbits. **(A)** Lung weight/body weight (LW/BW) ratios, and **(B–D)** bacterial burden in lung, spleen and kidneys increased over time and peaked at the time of death in the 36 rabbits that were challenged by endobronchial instillation with 1.1 x 10^8^ CFU of live Pa6206. Of these, 30 Pa6206-challenged rabbits were euthanized at predetermined time points of 3, 4, 5, 6, 10 h post-infection (6 rabbits for each time point). The remaining 6 live Pa6206-challenged rabbits were euthanized when they exhibited signs of respiratory distress and had blood lactate >10 mmol/L—their terminal endpoints (TE) were between 13 and 23 h post-infection. Seven control control rabbits were included for comparison: 1 control rabbit was euthanized at 5 h after endobronchial instillation with lactated Ringer’s solution (LRS); and 3 control rabbits each were euthanized at 5 h or 96 h, respectively, after endobronchial instillation with UV-killed Pa6206 (6.6 x 10^8^ CFUs irradiated by ultraviolet light for 30 minutes). No bacteria were cultured from lung, spleen and kidneys from the seven control rabbits.

The extent of acute lung inflammation and pulmonary edema over time was evaluated quantitatively by changes in LW/BW ratios. Compared to LW/BW of 4.6 ± 0.4 g/kg (mean ± standard deviation) for control rabbits instilled endobronchially with LRS or UV-killed Pa6206, these ratios significantly increased over time for rabbits challenged with live Pa6206 (*P*<0.001 by test for linear trend), peaking at 14.3 ± 1.0 g/kg at the terminal endpoint (**Figure 3A**). The LW/BW ratios exceeded 10 g/kg, a threshold value that was previously associated with profound respiratory failure and death in a rabbit non-ventilated pneumonia model (Le et al., 2018), for 83% (5/6) of rabbits in the 10 h post-infection group and 100% (6/6) of those in the terminal endpoint group (**Figure 3A**). In contrast, at 5 and 6 h post-infection, only one rabbit in each group had LW/BW >10 g/kg (10.68 and 10.71, respectively), suggesting that antibacterial intervention administered at these earlier time points could still halt disease progression. The progressive increase in LW/BW was due to pulmonary infection and not endotoxemia *per se*, as indicated by growth of Pa6206 in the lungs over time (*P*<0.001 by test for linear trend) (**Figure 3B**), reaching maximum bacterial density of log_10_CFU of 10.09 ± 0.49 (mean ± standard deviation) at the terminal endpoint when rabbits developed respiratory failure, which is >100-fold increase bacterial density compared to 1.1 x 10^8^ CFUs of live Pa6206 used for endobronchial challenge. The extent of the infection was also evidenced by systemic dissemination of bacteria from lung to other distal organs, including spleen and kidneys, that peaked at the terminal endpoint (*P*<0.001 by test for linear trend) (**Figures 3C, D**). In comparison, only 50% (3/6) of rabbits at 5 h post-infection and 6 h post-infection had disseminated infection.

**Figure 4:**
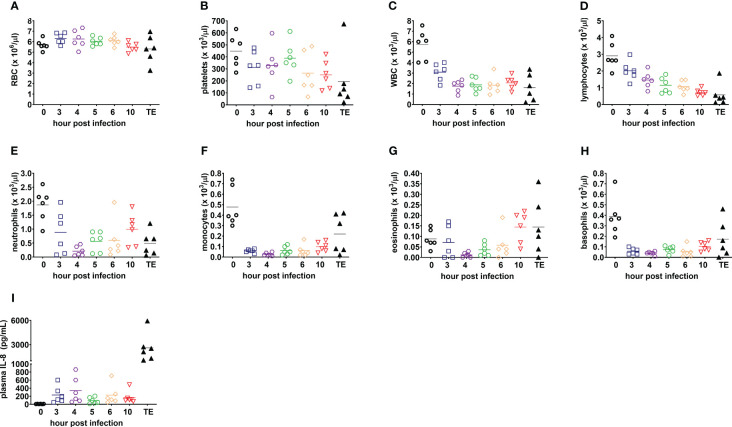
Blood counts and plasma IL-8 are consistent with acute respiratory distress syndrome and sepsis in the rabbit natural history study. **(A)** Red blood cells, RBC; **(B)** platelets; **(C)** white blood cells, WBC; **(D)** lymphocytes; **(E)** neutrophils; **(F)** monocytes; **(G)** eosinophils; **(H)** basophils; and **(I)** plasma interleukin-8, IL-8 (CXCL-8). Linear test for trend was used to evaluate whether means for each parameter increase (or decrease) systematically over the seven sampling time points, namely, pre-infection (0), 3, 4, 5, 6, and 10 h post-infection, and terminal endpoint between 13 and 23 h post-infection when rabbits were euthanized for development of respiratory failure when their lactate exceeded 10 mmol/L.

Deranged hematological parameters were also observed in infected rabbits (**Figure 4**). Severe thrombocytopenia was noted in 83% (5/6) of rabbits at the terminal endpoint (**Figure 4B**). Severe neutropenia occurred in all infected rabbits as early as 4 hpi and was correlated with elevated levels of the neutrophil chemoattractant, interleukin 8 (IL-8, CXCL-8) (**Figures 4E, I**) (Thompson et al., 2017).

**Figure 5:**
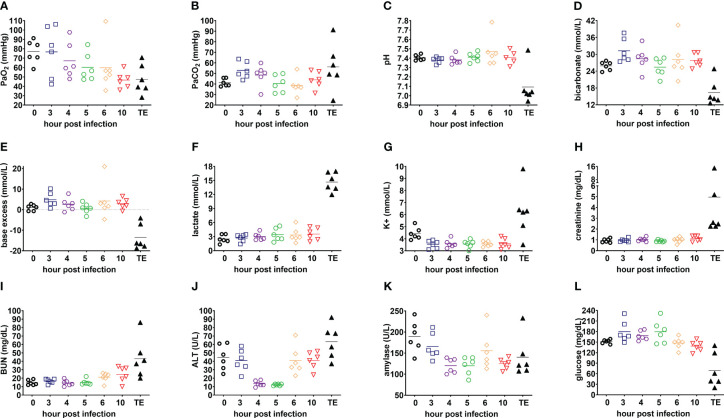
Evolution of clinically relevant biomarkers of acute respiratory distress syndrome in the rabbit natural history study. **(A)** PaO_2_; **(B)** PaCO_2_; **(C)** pH; **(D)** bicarbonate; **(E)** base excess; **(F)** lactate; **(G)** potassium, K^+^; **(H)** creatinine; **(I)** blood urea nitrogen, BUN; **(J)** alanine aminotransferase, ALT; **(K)** amylase; and **(L)** glucose. Linear test for trend was used to evaluate whether means for each parameter increase (or decrease) systematically over the seven sampling time points, namely, pre-infection (0), 3, 4, 5, 6, and 10 h post-infection, and terminal endpoint between 13 and 23 h post-infection when rabbits were euthanized for development of respiratory failure when their lactate exceeded 10 mmol/L.

Blood gas values were progressively altered, with PaO_2_<60mmHg while breathing room air observed for 67% (4/6) of infected rabbits at 5 h post-infection and 83% (5/6) at 6 h post-infection and decreased further at 10 h post infection and terminal endpoint (**Figure 5A**). Blood lactate levels peaked at the terminal endpoint (>10 mmol/L; **Figure 5F**), which is consistent with this being a prognostic biomarker of increased mortality in patients with ARDS (Hack et al., 1992; Casserly et al., 2015; Singer et al., 2016; Kamo et al., 2019). Respiratory failure was associated with acidosis at terminal endpoint (**Figures 5B–E**) (Berend et al., 2014). Potassium, creatinine, blood urea nitrogen (BUN), alanine aminotransferase (ALT) and amylase (**Figures 5G–K**) peaked at the terminal endpoint, consistent with an evolution toward ARDS-associated multiple organ failure. Severe hypoglycemia (defined as <60 mg/dL compared to base line of 140-160 mg/dL) was noted in 67% (4/6) of infected rabbits at the terminal endpoint (**Figure 5L**), which mimics altered glycemic control observed in patients with Gram-negative sepsis (Peralta et al., 2010; Levit et al., 2014).

Taken together, the 5 h post-infection time point may be suitable for initiation of antibacterial intervention because lung histopathological analysis showed evidence of pneumonia onset, with mild to moderate diffuse PMN infiltrates, multifocal altered PMN aggregates, alveolar hemorrhage (**Table 1**) and hypoxemia (PaO_2_ < 60mmHg in **Figure 5A**) in a majority of infected rabbits.

### Antibiotic treatment in a rabbit *P. aeruginosa* non-ventilated pneumonia model

An FDA-recommended strategy for animal model validation is to evaluate whether antibiotics with known efficacy for treating human pneumonia would yield similar efficacy in the rabbit non-ventilated pneumonia model (Byrne et al., 2020). A potential challenge with this strategy is the selection of appropriate antibiotic dose and dosing interval in the animal model that would mimic clinical PK-PD targets in humans. As such, we performed PK studies in uninfected rabbits to select the human-equivalent dose and dosing intervals (**Tables 2**, **3**) and then evaluated efficacy of the humanized dosing regimens in the rabbit non-ventilated pneumonia model.

**Table 2:** Pharmacokinetics of tobramycin in New Zealand white rabbits.

	2.5 mg/kg IV bolus in uninfected rabbits	2.5 mg/kg IV bolus in infected rabbits	5.0 mg/kg IV bolus in infected rabbits
t_1/2_, h	2.68	2.63	4.30
Vss, (mg/kg)/(μg/ml)	0.28	0.29	0.57
CL, (mg/kg)/(μg/ml)/h	0.07	0.08	0.09
AUC_0-8h_, μg/ml*h	29.8	28.6	39.5
C_max_/MIC at 0.5 hpd	18.8	18.2	15.2
C_min_/MIC at 8 hpd	0.39	0.12	0.12

Pa6206 tobramycin MIC: 1.0 µg/mL; t_1/2_, half-life; Vss, volume of distribution at steady state; CL, clearance; AUC_0-8h_, area under the plasma concentration-time curve in 0-8 h post dose (hpd); peak concentration [C_max_] to MIC at 0.5 hpd; trough concentration [C_min_] to MIC at 8 hpd.

**Table 3:** Pharmacokinetics of meropenem in New Zealand white rabbits.

	80 mg/kg IV bolus in uninfected rabbits	80 mg/kg IV bolus in infected rabbits
t_1/2_, h	0.54	0.66
Vss, (mg/kg)/(μg/ml)	0.96	1.01
CL, (mg/kg)/(μg/ml)/h	1.23	1.06
AUC_0-2h_, μg/ml*h	60.0	66.0
AUC_0-8h_, μg/ml*h	240.0	263.9
C_max_/MIC at 0.5 hpd	175.0	218.1
C_min_/MIC at 2.0 hpd	3.38	5.82

Pa6206 meropenem MIC: 0.25 µg/mL; t_1/2_, half-life; Vss, volume of distribution at steady state; CL, clearance; AUC_0-8h_, area under the plasma concentration-time curve in 0-8 h post dose (hpd); peak concentration [C_max_] to MIC at 0.5 hpd; trough concentration [C_min_] to MIC at 2 hpd.

**Figure 6:**
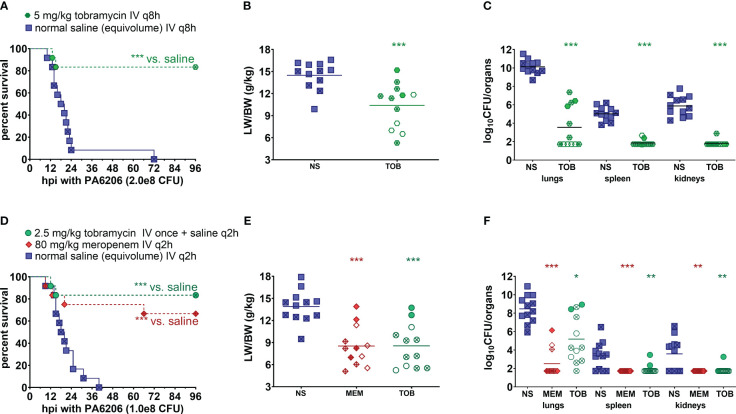
Human-equivalent meropenem and tobramycin dosing regimens protected against lethal infection in the rabbit non-ventilated pneumonia model with *P. aeruginosa*. **(A, D)** Kaplan-Meier survival curves, **(B, E)** LW/BW ratio, and **(C, F)** log_10_(CFU/organ). For the first validation study **(A–C)**, rabbits were randomized at 5 h post-infection with 2.0 x 10^8^ CFU Pa6206 for treatment with normal saline (NS; 0.9% NaCl) IV q8h or tobramycin (TOB) 5 mg/kg IV q8h at 5 h post-infection (12 rabbits per experimental group). For the second validation study **(D-F)**, rabbits were randomized at 5 h post infection (h post-infection) with 1.0 x 10^8^ CFU Pa6206 for treatment with normal saline IV q2h, meropenem (MEM) 80 mg/kg IV q2h, or tobramycin (TOB) 2.5 mg/kg IV once at 5 h post-infection and then normal saline IV q2h (12 rabbits per experimental group). One rabbit in the meropenem group died at 9 h post-infection, which was about 3 minutes after IV injection of the second dose of meropenem; this rabbit did not show any sign of respiratory distress sepsis or respiratory failure immediately prior to antibiotic injection. One-sided log-rank (Mantel-Cox) test was used to test the hypothesis that survival of animals treated with saline is shorter than survival of those treated with meropenem or tobramycin, using *P*<0.025 (significance level of 0.05 divided by two different comparisons) being considered statistically significant to account for multiple comparisons using Bonferroni method. LW/BW ratio and bacterial densities for saline-treated animals were compared to those of each of the other two treatment groups by nonparametric one-way analysis of variance (ANOVA) with Kruskal-Wallis test followed by Dunn’s multiple comparisons test. Filled symbols represent data from dead animals, and open symbols represent data from surviving animals that were euthanized at 96 h post-infection. X-marked symbols represent data from male rabbits, whereas those not marked with an X represent data from female rabbits. Statistical significance abbreviations: * for *P*<0.05; ** for *P*<0.01; *** for *P*<0.001.

In the first validation study, rabbits were randomized for treatment with humanized tobramycin regimen of 5 mg/kg q8h (3 doses total for 24 h of drug exposure) or an equivalent volume of saline q8h. For tobramycin which exhibits concentration-dependent killing, C_max_/MIC is one of the most important parameters predictive of its efficacy, with C_max_/MIC ≥ 10 associated with the clinical therapeutic response in patients with Gram-negative pneumonia (Moore et al., 1987; Kashuba et al., 1998). To mimic the efficacious 7-8 mg/kg/day tobramycin dosing regimen in humans, we administered a humanized dosing regimen of 5 mg/kg tobramycin q8h at 5 h post-infection, which yielded C_max_ of 9.8 ± 1.4 µg/mL at 60 min post-dose and C_min_ of 0.12 ± 0.01 µg/mL at 8 h post dose (**Table 2**). Mortality rates were 100% (12/12) for saline-treated rabbits, compared to 17% (2/12) for tobramycin-treated rabbits (*P*<0.001 by log-rank test) (**Figure 6A**). LW/BW ratios, a quantitative marker of acute lung injury and pulmonary edema, were significantly reduced for tobramycin-treated rabbits compared to those treated with saline (**Figure 6B**). Bacterial counts in lungs, spleen and kidneys were significantly reduced for tobramycin-treated rabbits compared to saline-treated rabbits, with microbiological clearance below the limit of detection achieved in 50% (6/12) of lungs harvested from tobramycin-treated rabbits (**Figure 6C**).

In the second validation study, rabbits were randomized for treatment at 5 h post-infection with a lesser dosing regimen of 2.5 mg/kg tobramycin (1 drug injection and then equivalent volume of saline q2h thereafter) or meropenem 80 mg/kg q2h or saline q2h. The 2.5 mg/kg tobramycin dosing regimen yielded C_max_ of 7.8 ± 0.8 µg/mL but C_min_ was below the limit of detection by 6 h post-dose (**Table 2**).

For meropenem which exhibits time-dependent bactericidal activity, % T>MIC and the ratio of minimum drug concentration to MIC (C_min_/MIC) are the most important parameters for predicting efficacy (Nicolau, 2008). To mimic the efficacious meropenem dosing regimen in humans, we administered humanized dosing regimen of 80 mg/kg q2h to achieve 100% T>MIC as well as C_min_/MIC of 3.4 for uninfected rabbits and 5.8 for infected rabbits (**Table 3**)—these values are in range with C_min_/MIC >5, which had previously been shown to correlate with clinical and microbiological response in 90% of patients with lower respiratory tract infections (Li et al., 2007). Because 100% T>MIC for meropenem is achievable in the vast majority of patients infected with drug-susceptible bacteria with MIC=0.25 µg/mL (Lodise et al., 2006), our humanized meropenem dosing regimen will reproduce this level of target attainment against Pa6206 with MIC=0.25 µg/mL. It was necessary to use a higher and more frequent humanized meropenem dosing regimen (compared to 1g q8h in humans) because of the very short half-life of 0.54-0.66 h in rabbits, although area under the concentration-time curve from 0 to 8 h (AUC_0–8h_, µg·h/ml) of 240.0 for uninfected rabbits and 263.9 for infected rabbits (**Table 3**) are similar to 234.79-282.35 for critically ill patients receiving intermittent bolus dosing of meropenem (Jamal et al., 2015).

Mortality rates were 100% (12/12) for saline-treated rabbits, compared to 17% (2/12) for rabbits treated with tobramycin 2.5 mg/kg once (*P*<0.001 vs. saline by log-rank test) and 33% (4/12) for those treated with meropenem 80 mg/kg q2h (*P*<0.001 vs. saline by log-rank test) (**Figure 6D**). LW/BW ratios were similar for meropenem and tobramycin-treated rabbits (*P*=1.00) but significantly reduced when compared to saline-treated rabbits (*P*<0.001) (**Figure 6E**). Bacterial counts in lungs, spleen and kidneys were significantly reduced in lungs of rabbits treated with meropenem or tobramycin when compared to saline-treated rabbits (*P*<0.001) (**Figure 6F**). Although the single dose of 2.5 mg/kg tobramycin protected rabbits against death, it was not sufficient to achieve microbiological clearance to below the limit of detection as only 8% (1/12) of treated rabbits had no bacteria in the lungs (**Figure 6F**) compared to 50% (6/12) of rabbits treated with tobramycin 5 mg/kg q8h (**Figure 6C**). Of the rabbits treated with the humanized meropenem dosing regimen, 75% (9/12) achieved microbiological clearance in lungs (**Figure 6F**).

To account for sex as a biological variable, individual data points from male rabbits were marked with an X whereas those from female rabbits were not marked (**Figure 6**). Male and female rabbits did not differ significantly in their response to meropenem or tobramycin treatment with regard to survival, LW/BW, or bacterial counts (**Figure 6**).

## Discussion

The urgent need to develop new therapeutic strategies against multidrug resistant bacteria, including *P. aeruginosa, A. baumannii* and *K. pneumoniae*, requires the development of well-validated animal models that could predict the human clinical response. We describe here the development and validation of a rabbit non-ventilated pneumonia model with *P. aeruginosa* by conducting (i) a natural history study of the infection to characterize whether pathophysiology in the infected animals reproduces pathophysiology in humans and to identify the appropriate timing for therapeutic intervention, and (ii) efficacy studies evaluating whether antibacterial drugs with known activities in humans also demonstrate similar efficacy in the animals.

In the rabbit non-ventilated pneumonia model, endobronchial challenge with live *Pa*6206, but not with UV-killed *Pa*6206, caused bilateral pneumonia, resulting in acute respiratory distress syndrome, respiratory failure and death between 13 to 23 h post-infection, with systemic dissemination of bacteria to multiple organs, including spleen and kidneys (**Table 1**; **Figures 2** **–** **6**). Hematogenous dissemination of bacteria is also found in 10-30% of pneumonias in humans, and associated with disease severity and poor outcomes (Paganin et al., 2004; Koulenti et al., 2017). As in humans, our model showed neutropenia and elevated IL-8 (CXCL-8) levels (Thommasen et al., 1984), which resulted in massive neutrophil recruitment into the lungs as shown through longitudinal histopathological analysis and consistent with the exudative phase of clinical acute respiratory distress syndrome (Antcliffe et al., 2018).

With a better understanding of pathophysiology in the rabbit non-ventilated pneumonia model, we were able to define clinically relevant triggers for initiating antibiotic treatment. At 5 h post-infection, two thirds of the rabbits had PaO_2_<60 mmHg (**Figure 5A**), corresponding to a cut-off when oxygen therapy would be required in the clinical setting and is considered as a marker of disease severity (Singer et al., 2016). When administered at 5 h post-infection, 100% of saline-treated rabbits developed lethal pneumonia compared to 17-33% of those treated with humanized dosing regimens of tobramycin and meropenem (**Figures 6A, D**), which are similar to mortality rates in patients with pneumonia (Paganin et al., 2004; Cilloniz et al., 2013; Koulenti et al., 2017). We evaluated efficacy of tobramycin dosed at 5 mg/kg q8h (x 3 doses) and at 2.5 mg/kg once, and found that 50% (6/12) and 8% (1/12) of rabbits in the respective groups achieved microbiological clearance, indicating that this model is capable of detecting dose-response relationship. Although tobramycin is not used alone clinically, this drug was tested alone in the rabbit model to establish its baseline efficacy in the absence of other antibiotics. Concomitant use of aminoglycoside is a major confounding factor in interpretation of clinical trial results for gram-negative infection, making it prudent in future preclinical studies to evaluate efficacy of new antibacterial drug candidate with and without tobramycin. The baseline efficacy data with tobramycin alone in our rabbit non-ventilated pneumonia model with *P. aeruginosa* (**Figure 6**) will be useful for enabling future combination drug studies.

Although we showed here that a humanized meropenem dosing regimen that achieved 100% T>MIC was protective in the rabbit non-ventilated pneumonia model against Pa6206 with MIC=0.25 µg/mL, we have previously shown in this same model that treatment failure occurred when subtherapeutic concentrations of this drug was used against *P. aeruginosa* strain 6077 with MIC=1.0 µg/mL (Le et al., 2019).

Development of both the rabbit model of non-ventilated pneumonia and the rabbit model of ventilator-associated pneumonia were supported under the same FDA contract (Byrne et al., 2020) because they reproduced different aspects of non-ventilated and ventilated pneumonia in humans (Zilberberg et al., 2022). In the rabbit model of non-ventilated pneumonia, the vast majority (28/30) of untreated or saline-treated rabbits had LW/BW >10 g/kg (**Figures 3A**, **6B, E**), a threshold value of severe pulmonary edema and acute hypoxemic respiratory failure (**Figure 5A**) (Le et al., 2018). In comparison, in the rabbit model of ventilator-associated pneumonia in which rabbits were ventilated with low-tidal volume of 6-7 mL/kg and PEEP of 5 cmH_2_O (Nguyen et al., 2021), only 54% (13/24) of untreated or saline-treated rabbits had LW/BW >10 g/kg; the remaining 46% (11/24) rabbits had LW/BW <10 g/kg but also succumbed to acute respiratory distress syndrome (Nguyen et al., 2021). Similar to ventilated hospital-associated bacterial pneumonia and ventilator-associated bacterial pneumonia in which 32.3% and 18.8% patients developed septic shock and required vasopressors (Zilberberg et al., 2022), the rabbits used in the ventilator-associated pneumonia model also developed severe hypotension and septic shock that were non-responsive to fluid challenge alone and required norepinephrine to maintain arterial blood pressure (Nguyen et al., 2021). The severe hypotension and septic shock observed in the rabbit model of ventilator-associated pneumonia required that treatment be administered earlier at 3 h post-infection (compared to the 5 h post-infection for the rabbit model of non-ventilated pneumonia, **Figure 6**) and that fluid challenge and norepinephrine be used to counteract lethal hypotension and allowed time for antibiotic treatment to exert its antimicrobial effects (Nguyen et al., 2021). In contrast, efficacy of antimicrobial agents can be tested in the rabbit model of non-ventilated pneumonia as described here (**Figure 6**) without the need for mechanical ventilation, fluid challenge, and norepinephrine, thereby reducing costs to the study sponsors.

Our rabbit non-ventilated pneumonia model with *P. aeruginosa* has limitations. First, we cannot determine based on the natural history study (**Figures 1** **–** **5**) whether rabbits died of profound hypoxemia or severe hypotension resulting from septic shock in the rabbit non-ventilated *P. aeruginosa* pneumonia model. More importantly, treatment of hypoxemia with mechanical ventilation or hypovolemic shock with fluid resuscitation and vasopressor therapy cannot be implemented in the non-ventilated model and better suited for the rabbit ventilator-associated pneumonia model (Nguyen et al., 2021). Second, antibacterial treatment was performed through bolus infusions whereas some data suggest that extended or continuous infusions may improve outcomes in humans (Roberts et al., 2009). Third, antibiotic treatment was triggered at a predetermined time point at 5 h post-infection, which was shown in our natural history study to coincide with pneumonia onset, but this was not triggered on a *per* animal basis based on derangement of biomarkers (e.g. treatment could be triggered when rabbits developed hypoxemia when PaO_2_<60 mmHg although this may not be experimentally feasible because blood collection for blood gas analysis are relatively difficult in the ambulatory rabbits and could delay the start of treatment). In any case, it may not be necessary to trigger antibacterial treatment on a *per* animal basis because the majority (4/6) of rabbits developed hypoxemia at 5 h post-infection (**Figure 5A**) and histopathological analyses strongly support pneumonia onset at this time point for all infected animals in the rabbit natural history study (**Figure 2**; **Table 1**). Fourth, dosing of meropenem is very different from rabbits to humans (80 mg/kg q2h compared to 1-2 g q8h, respectively) (Kalil et al., 2016). This is explained by a much shorter meropenem half-life of 0.54-0.66 h in rabbits compared to 1 h in humans, although meropenem AUC_0-8h_ in rabbits and in humans are similar (**Table 3**) (Jamal et al., 2015). Fifth, rabbits had only 24-h of antibiotic exposure. They were then monitored for survival for an additional 67 h during which bacteria that survived the 24-h antibiotic exposure period could rebound and cause lethal infection. We observed only a single meropenem-treated rabbit that died at 66 h post-infection during the post-antibiotic exposure period (**Figure 6D**), but curiously this rabbit had no bacteria cultured from its lung, spleen or kidneys, and its LW/BW was 6.98 (**Figures 6E, F**) so its cause of death is unclear. During the post-antibiotic exposure period, the host immune system could also impact bacterial burden in the infected organs, thereby complicating interpretations of CFU counts in the various experimental groups. As such, we have made a tradeoff of prioritizing survival, a more clinically relevant experimental outcome, over CFU counts in our rabbit non-ventilated pneumonia model. This model, however, is amendable to having CFU counts (or even the degree of acute lung injury, i.e., LW/BW) as the primary experimental outcome in antibiotic treatment studies by using a sublethal bacterial challenge dose and euthanizing rabbits at the end of the antibiotic exposure period so that bacterial count can be determined at the same time for all animals in various experimental arms. Also, our humanized meropenem dosing regimen of 80 mg/kg q2h (12 intravenous doses over a 24-h period) is the maximum approved for our animal protocol because the rabbits are unlikely to tolerate additional stress from such frequent dosing for another 24-h or 48-h of drug dosing. Finally, although we reported herein an expansive dataset for a single Pa6206 strain, the rabbit non-ventilated pneumonia model was also used successfully with another *P. aeruginosa* 6077 strain (Le et al., 2018) as well as numerous methicillin-resistant *Staphylococcus aureus* strains (Diep et al., 2010; Diep et al., 2013; Diep et al., 2016; Diep et al., 2017; Vu et al., 2019; Tran et al., 2020).

In conclusion, well-characterized animal models are urgently needed to evaluate the activity of new antibacterial drugs. Under a broad framework proposed for animal model development and validation (Byrne et al., 2020; Waack et al., 2020), we have characterized and validated a rabbit non-ventilated pneumonia model with *P. aeruginosa* that could be used to help predict clinical efficacy of new antibacterial drugs.

## Data availability statement

The raw data supporting the conclusions of this article will be made available by the authors, without undue reservation.

## Ethics statement

The animal study was approved by University of California San Francisco Institutional Animal Care and Use Committee. The study was conducted in accordance with the local legislation and institutional requirements.

## Author contributions

EG: Data curation, Formal analysis, Investigation, Methodology, Visualization, Writing – review & editing. TV: Data curation, Formal analysis, Investigation, Writing – review & editing. NN: Data curation, Investigation, Writing – review & editing. VT: Investigation, Writing – review & editing. YM: Investigation, Writing – review & editing. NT: Investigation, Writing – review & editing. NM: Investigation, Writing – review & editing. OD: Investigation, Writing – review & editing. DJ: Investigation, Writing – review & editing. NI: Investigation, Writing – review & editing. HP: Investigation, Writing – review & editing. MP: Investigation, Writing – review & editing. FA-A: Investigation, Writing – review & editing. WW: Investigation, Writing – review & editing, Methodology. BZ: Methodology, Writing – review & editing. LC: Methodology, Writing – review & editing. CS: Methodology, Writing – review & editing. BS: Methodology, Writing – review & editing. AD: Methodology, Writing – review & editing. LG: Methodology, Writing – review & editing, Investigation. FV: Investigation, Methodology, Writing – review & editing. BD: Investigation, Methodology, Conceptualization, Data curation, Formal analysis, Funding acquisition, Project administration, Resources, Supervision, Visualization, Writing – original draft.
